# The Unique Range Set of Meromorphic Functions in an Angular Domain

**DOI:** 10.1155/2014/564640

**Published:** 2014-05-15

**Authors:** Hong-Yan Xu, Zu-Xing Xuan, Hua Wang

**Affiliations:** ^1^Department of Informatics and Engineering, Jingdezhen Ceramic Institute, Jingdezhen, Jiangxi 333403, China; ^2^Basic Department, Beijing Union University, No. 97 Bei Si Huan Dong Road, Chaoyang District, Beijing 100101, China

## Abstract

By using Tsuji's characteristic, we investigate uniqueness of meromorphic functions in an angular domain dealing with the shared set, which is different from the set of the paper (Lin et al., 2006) and obtain a series of results about the unique range set of meromorphic functions in angular domain.

## 1. Introduction

The purpose of this paper is to deal with the uniqueness problem of meromorphic functions sharing one set in an angular domain by using Tsuji's characteristic. Thus, the notation and theory of Nevanlinna (see [1, 2]) about meromorphic function are basis for readers.

We use *C* to denote the open complex plane, $\hat{C}\;\;\left(=C{⋃}_{}\left\{\infty \right\}\right)$ to denote the extended complex plane, and *Ω*(⊂*C*) to denote an angular domain.

In 1929, Nevanlinna (see [3]) first investigated the uniqueness of meromorphic functions in the whole complex plane and obtained the well-known theorem-5 *IM* theorem of two meromorphic functions sharing five distinct values.

**Theorem 1 (see [3])**

If *f* and *g* are two nonconstant meromorphic functions that share five distinct values *a*_1_, *a*_2_, *a*_3_, *a*_4_, and *a*_5_ *IM* in *C*, then *f*(*z*) ≡ *g*(*z*).

After his theorems, the uniqueness problems of meromorphic functions sharing values in the whole complex plane attracted many investigations (see [2]). In 2004, Zheng [4] studied the uniqueness problem under the condition that five values are shared in some angular domain in *C*. In recent years, there are many results on the uniqueness of meromorphic function in an angular domain sharing values and sets (see [5–16]). Zhang [15], Zheng [17], Cao and Yi [18], Xu and Yi [19], and Xuan [20] continued to investigate the uniqueness of meromorphic functions sharing five values and four values, and Lin et al. [8] and Lin et al. [7] investigated the uniqueness of meromorphic and entire functions sharing sets in an angular domain. To state their results, we need the following basic notations and definitions of meromorphic functions in an angular domain (see [1, 4, 17]).

Let *S* be a set of distinct elements in $\hat{C}$ and *Ω*⊆*C*. Define

(1) E(S,Ω,f)  =⋃a∈S{z∈Ω ∣ fa(z)=0,  counting  multiplicities},E¯(S,Ω,f)  =⋃a∈S{z∈Ω ∣ fa(z)=0,  ignoring  multiplicities},

where *f*_*a*_(*z*) = *f*(*z*) − *a* if *a* ∈ *C* and *f*_*∞*_(*z*) = 1/*f*(*z*). We also define

(2) $$\begin{array}{c} E_{1}\left(S,\Omega ,f\right)=\underset{a\in S}{⋃}\left\{z\in \Omega :\;\text{all}\;\text{the}\;\text{simple}\;\text{zeros}\;\text{of}\;f_{a}\left(z\right)\right\}. \end{array}$$

Let *f* and *g* be two nonconstant meromorphic functions in *C*. If *E*(*S*, *Ω*, *f*) = *E*(*S*, *Ω*, *g*), we say *f* and *g* share the set *S* *CM* (counting multiplicities) in *Ω*. If $\bar{E}\left(S,\Omega ,f\right)=\bar{E}\left(S,\Omega ,g\right)$, we say *f* and *g* share the set *S* *IM* (ignoring multiplicities) in *Ω*. In particular, when *S* = {*a*}, where $a\in \hat{C}$, we say *f* and *g* share the value *a* *CM* in *Ω* if *E*(*S*, *Ω*, *f*) = *E*(*S*, *Ω*, *g*), and we say *f* and *g* share the value *a* *IM* in *Ω* if $\bar{E}\left(S,\Omega ,f\right)=\bar{E}\left(S,\Omega ,g\right)$. When *Ω* = *C*, we give the simple notation as before, $E\left(S,f\right),\bar{E}\left(S,f\right)$, and so on (see [19]).

In 2006, Lin et al. [7] dealt with the uniqueness problem on meromorphic functions sharing three finite sets in an angular domain and obtained the following theorems.

**Theorem 2 (see [7, Thereom 1])**

Let *S*_1_ = {*∞*}, *S*_2_ = {*ω* | *ω* ^*n*−1^(*ω* + *a*) − *b* = 0}, and *S*_3_ = {0}, where *n*(≥4) is an integer and *a*, *b* are two nonzero constants, such that the algebraic equation *ω* ^*n*−1^(*ω* + *a*) − *b* = 0 has no multiple roots. Assume that *f* is a meromorphic function of lower order *μ*(*f*) ∈ (1/2, *∞*) in $\hat{C}$ and *δ* : = *δ*(*ι*, *f*) > 0 for some $\iota \in \hat{C}\setminus \left\{0,-a\right\}$. Then, for each *σ* < *∞* with *μ*(*f*) ≤ *σ* ≤ *λ*(*f*), there exists an angular domain *Ω* = *Ω*(*α*, *β*) : = {*z* : *α* < arg⁡*z* < *β*} with 0 ≤ *α* < *β* ≤ 2*π* and

(3) $$\begin{array}{c} \beta -\alpha >\max\left\{\frac{\pi }{\sigma },2\pi -\frac{4}{\sigma }\arcsin\sqrt{\frac{\delta }{2}}\right\} \end{array}$$

such that if the conditions *E*(*S*_3_, *f*) = *E*(*S*_3_, *g*) and *E*(*S*_*j*_, *Ω*, *f*) = *E*(*S*_*j*_, *Ω*, *g*)  (*j* = 1,2) hold for a meromorphic function *g* of finite order or, more generally, with the growth satisfying either log⁡*T*(*r*, *g*) = *O*(log⁡*T*(*r*, *f*)) or

(4) $$\begin{array}{c} \underset{r\to \infty }{\lim}\frac{\log\;\log T\left(r,g\right)}{\min\left\{\log r,\log T\left(r,f\right)\right\}}=0,\;r\notin E_{1}, \end{array}$$

where *E*_1_ is a set of finite linear measures, then *f* ≡ *g*.

In 2011, Chen and Lin [21] further investigated the uniqueness of meromorphic functions sharing three finite sets in an angular domain and obtained the following result.

**Theorem 3 (see [21, Thereom 1])**

Let *S*_1_ and *S*_2_ be defined as in Theorem 2, and let *n* ≥ 8 be an integer. Assume that *f* is a meromorphic function of lower order *μ*(*f*) ∈ (1/2, *∞*) in *C* and Θ(*∞*, *f*) > 2/(*n* − 1) and that *g* is a meromorphic function of finite order or, more generally, with the growth satisfying either log⁡*T*(*r*, *g*) = *O*(log⁡*T*(*r*, *f*)) or condition (4). Then, for each *σ* < *∞* with *μ*(*f*) ≤ *σ* ≤ *λ*(*f*), there exists an angular domain *Ω* = *Ω*(*α*, *β*) with 0 ≤ *α* < *β* ≤ 2*π* and condition (3), such that if *E*(*S*_*j*_, *Ω*, *f*) = *E*(*S*_*j*_, *Ω*, *g*)  (*j* = 1,2), then *f* ≡ *g*.

In 2010, Zheng [16] proved the following theorem by using Tsuji's characteristic to extend the five-*IM* theorem of Nevanlinna's to an angular domain. Tsuji's characteristic will be introduced in Section 2.

**Theorem 4 (see [16])**

Let *f*(*z*) and *g*(*z*) be both meromorphic functions in an angular domain *Ω* = {*z* : *α* < arg⁡*z* < *β*} with 0 ≤ *α* < *β* ≤ 2*π*, and let *f*(*z*) be transcendental in Tsuji's sense. Assume that *a*_*j*_  (*j* = 1,2,…, 5) are 5 distinct complex numbers. If $\bar{E}\left(a_{j},\Omega ,f\right)=\bar{E}\left(a_{j},\Omega ,g\right)$, then *f*(*z*) ≡ *g*(*z*).

## 2. Main Results

In this paper, we will focus on the uniqueness problem of shared set of meromorphic functions in an angular domain by using Tsuji's characteristic. In fact, we will study the uniqueness of meromorphic functions in an angular domain sharing one set of the form *S* = {*w* ∈ *A* : *P*_1_(*w*) = 0}, where

(5) P1(w)=(n−1)(n−2)2wn−n(n−2)wn−1 +n(n−1)2wn−2−c,

and let *c* be a complex number satisfying *c* ≠ 0,1, and obtain the following results.

**Theorem 5**

Let *f*(*z*) and *g*(*z*) be both meromorphic functions in an angular domain *Ω* = {*z* : *α* < arg  *z* < *β*} with 0 ≤ *α* < *β* ≤ 2*π*, and let *f*(*z*) be transcendental in Tsuji's sense. If *E*(*S*, *Ω*, *f*) = *E*(*S*, *Ω*, *g*) and *n* is an integer ≥11, then *f* ≡ *g*.

A set *S* is called a unique range set for meromorphic functions in an angular domain *Ω*, if for any two nonconstant meromorphic functions *f* and *g* the condition *E*(*S*, *Ω*, *f*) = *E*(*S*, *Ω*, *g*) implies *f* ≡ *g*. We denote by *♯S* the cardinality of a set *S*. Thus, from Theorem 5, we can get the following corollary.

**Corollary 6**

There exists one finite set *S* with *♯S* = 11, such that any two meromorphic functions *f* and *g* in an angular domain *Ω* which are transcendental in Tsuji's sense must be identical if *E*(*S*, *Ω*, *f*) = *E*(*S*, *Ω*, *g*).

**Theorem 7**

Let *f*(*z*) and *g*(*z*) be both meromorphic functions in an angular domain *Ω* = {*z* : *α* < arg  *z* < *β*} with 0 ≤ *α* < *β* ≤ 2*π*, and let *f*(*z*) be transcendental in Tsuji's sense. If *E*(*S*, *Ω*, *f*) = *E*(*S*, *Ω*, *g*), Θ_*T*_(*∞*, *f*) > 3/4, Θ_*T*_(*∞*, *g*) > 3/4, and *n* is an integer ≥7, then *f* ≡ *g*.

**Corollary 8**

There exists one finite set *S* with *♯S* = 7, such that any two analytic functions *f* and *g* in *Ω* which are transcendental in Tsuji sense must be identical if *E*_1_(*S*, *Ω*, *f*) = *E*_1_(*S*, *Ω*, *g*).

**Theorem 9**

Let *f*(*z*) and *g*(*z*) be both meromorphic functions in an angular domain *Ω* = {*z* : *α* < arg⁡*z* < *β*} with 0 ≤ *α* < *β* ≤ 2*π*, and let *f*(*z*) be transcendental in Tsuji's sense. If *E*_1_(*S*, *Ω*, *f*) = *E*_1_(*S*, *Ω*, *g*) and *n* is an integer ≥15, then *f* ≡ *g*.

A set *S* is called a unique range set with weight 1 for meromorphic functions in *Ω*, if for any two nonconstant meromorphic functions *f* and *g* the condition *E*_1_(*S*, *Ω*, *f*) = *E*_1_(*S*, *Ω*, *g*) implies *f* ≡ *g*. Thus, from Theorem 9, we can get the following corollary.

**Corollary 10**

There exists one finite set *S* with *♯S* = 15, such that any two meromorphic functions *f* and *g* in an angular domain *Ω* which are transcendental in Tsuji's sense must be identical if *E*_1_(*S*, *Ω*, *f*) = *E*_1_(*S*, *Ω*, *g*).

**Theorem 11**

Let *f*(*z*) and *g*(*z*) be both meromorphic functions in an angular domain *Ω* = {*z* : *α* < arg⁡*z* < *β*} with 0 ≤ *α* < *β* ≤ 2*π*, and let *f*(*z*) be transcendental in Tsuji's sense. If *E*_1_(*S*, *Ω*, *f*) = *E*_1_(*S*, *Ω*, *g*), Θ_*T*_(*∞*, *f*) > 5/6, Θ_*T*_(*∞*, *g*) > 5/6, and *n* is an integer ≥9, then *f* ≡ *g*.

From Theorem 11, we can get the corollary as follows.

**Corollary 12**

There exists one finite set *S* with *♯S* = 9, such that any two analytic functions *f* and *g* in *Ω* which are transcendental in Tsuji sense must be identical if *E*_1_(*S*, *Ω*, *f*) = *E*_1_(*S*, *Ω*, *g*).

We found that the conclusions of Theorems 5–11 and Corollaries 6–12 hold for transcendental functions *f* in Tsuji sense.

Thus, a question arises naturally, whether the conclusions of these theorems and corollaries hold for a general function in an angular domain.

For the above question, we can get the following theorem.

**Theorem 13**

Let the assumptions of Theorems 5–11 and Corollaries 6–12 be given with the exception that *f*(*z*) is transcendental in Tsuji sense. Assume that, for some $a\in \hat{C}$ and *ε* > 0,

$$\begin{array}{cc} \left(‡\right) & \underset{r\to \infty }{\mathrm{limsup}}\frac{N\left(r,\Omega_{\varepsilon },f=a\right)}{r^{\omega }\log r}=\infty , \end{array}$$

where *ω* = *π*/(*β* − *α*), *N*(*t*, *Ω*, *f*) = ∫_1_^*r*^(*n*(*t*, *Ω*, *f*)/*t*)*dt*, and *n*(*t*, *Ω*, *f*) is the number of poles of *f*(*z*) in *Ω*∩{*z* : 1 < |*z*| ≤ *t*}. Then *f*(*z*) ≡ *g*(*z*).

## 3. Preliminaries and Some Lemmas

In this section, we will introduce some notations of Tsuji's characteristic in an angular domain (see [16, 22]). For meromorphic function *f* in an angular domain *Ω* and *ω* = *π*/(*β* − *α*), we define

(6) Mα,β(r,f) =12π∫arcsin⁡(r−ω)π−arcsin⁡(r−ω)log⁡+|f(rei(α+ω−1θ)sinω−1θ)|1rωsin2θ dθ,Nα,β(r,f) =∑1<|bn|<r(sin⁡(ω(βn−α)))ω−1(sinω(βn−α)|bn|ω−1rω),

where *b*_*n*_ = |*b*_*n*_|*e* ^*iβ*_*n*_^ are the poles of *f*(*z*) in *Ξ*(*α*, *β*; *r*) = {*z* = *re* ^*iθ*^ : *α* < *θ* < *β*, 1 < *t* ≤ *r*(sin⁡(*ω*(*β*_*n*_ − *α*)))^*ω*^−1^^} appearing often according to their multiplicities and then Tsuji characteristic of *f* is

(7) $$\begin{array}{c} T_{\alpha ,\beta }\left(r,f\right)=M_{\alpha ,\beta }\left(r,f\right)+N_{\alpha ,\beta }\left(r,f\right). \end{array}$$

We denote by *n*_*α*,*β*_(*r*, *f*) the number of poles of *f*(*z*) in *Ξ*(*α*, *β*; *r*), then

(8) Nα,β(r,f)=∫1r(1tω−1rω)dnα,β(t,f)=ω∫1rnα,β(t,f)tω+1 dt,

when pole *b*_*n*_ occurs in the sum ∑_1<|*b*_*n*_|<*r*(sin(*ω*(*β*_*n*_−*α*)))^*ω*^−1^^_ only once, and we denote it by ${\bar{N}}_{\alpha ,\beta }\left(r,f\right)$. For meromorphic function *f* in *Ω* and for all complex numbers *a*, if

(9) $$\begin{array}{c} \underset{r\to \infty }{\mathrm{limsup}}\frac{T_{\alpha ,\beta }\left(r,f\right)}{\log r}=\infty , \end{array}$$

then *f* is called transcendental with respect to the Tsuji characteristic [16], and we have the Tsuji deficiency of *f*(*z*) as follows:

(10) δT(a,f;α,β)=liminf⁡r→∞Mα,β(r,1/(f−a))Tα,β(r,f)=1−limsup⁡r→∞Nα,β(r,1/(f−a))Tα,β(r,f);ΘT(a,f;α,β)=1−limsup⁡r→∞N¯α,β(r,1/(f−a))Tα,β(r,f),

for *a*≢*∞*, *δ*_*T*_(*∞*, *f*; *α*, *β*) is defined by the above formula with *M*_*α*,*β*_(*r*, *f*) and *N*_*α*,*β*_(*r*, *f*) in place of *M*_*α*,*β*_(*r*, 1/(*f* − *a*)) and *N*_*α*,*β*_(*r*, 1/(*f* − *a*)), and Θ_*T*_(*∞*, *f*; *α*, *β*) is defined by the above formula with ${\bar{N}}_{\alpha ,\beta }\left(r,f\right)$ in place of ${\bar{N}}_{\alpha ,\beta }\left(r,1/\left(f-a\right)\right)$. If no confusion occurs in the context, then we simply write *δ*_*T*_(*a*, *f*) for *δ*_*T*_(*a*, *f*; *α*, *β*) and Θ_*T*_(*a*, *f*) for Θ_*T*_(*a*, *f*; *α*, *β*). *δ*_*T*_(*a*, *f*) is called the Tsuji deficiency of *f* at *a* and if *δ*_*T*_(*a*, *f*) > 0, then *a* is said to be a Tsuji deficient value of *f*. In addition, from [16], we have the following properties of this Tsuji's characteristic:

(11) $$\begin{array}{c} T_{\alpha ,\beta }\left(r,\frac{1}{f-a}\right)=T_{\alpha ,\beta }\left(r,f\right)+O\left(1\right), \end{array}$$

and from [16, Lemma 2.5.4], the fundamental inequalities

(12) $$\begin{array}{c} \left(q-2\right)T_{\alpha ,\beta }\left(r,f\right)\leq \overset{q}{\underset{j=1}{\sum }}{\bar{N}}_{\alpha ,\beta }\left(r,\frac{1}{f-a_{j}}\right)+Q_{\alpha ,\beta }\left(r,f\right) \end{array}$$

hold for *q* distinct points $a_{j}\in \hat{C}$,

(13) $$\begin{array}{c} Q_{\alpha ,\beta }\left(r,f\right)=O\left(\log^{+}T_{\alpha ,\beta }\left(r,f\right)+\log r\right),\;r\notin E, \end{array}$$

where *E* denotes a set of *r* with finite linear measure. It is not necessarily the same for every occurrence in the context.

**Remark 14**

In fact, from (12) and [16], we can get that the form

(14) (q−2)Tα,β(r,f) ≤∑j=1qN¯α,β(r,1f−aj)−Nα,β0(r,1f′)+Qα,β(r,f)

holds for *q* distinct points $a_{j}\in \hat{C}$, where *Q*_*α*,*β*_(*r*, *f*) satisfies (13) and *N*_*α*,*β*_^0^(*r*, 1/*f*′) is the counting function of the zeros of *f*′ in *Ω* where *f* does not take any of the values *a*_*j*_  (*j* = 1,2,…, *q*).

**Lemma 15 (see [16, Lemma 2.5.4])**

Assume that *f*(*z*) is a meromorphic function in *Ω*(*α*, *β*). Then for 0 < *r* < *R*, one has

(15) $$\begin{array}{c} M_{\alpha ,\beta }\left(r,\frac{f^{\left(p\right)}}{f}\right)\leq K\left[\log^{+}T_{\alpha ,\beta }\left(R,f\right)+\log\frac{R}{R-r}+1\right], \end{array}$$

where *K* is a constant independent of *r* and *R*.

For sake of simplicity, we omit the subscript in all notations and use *M*(*r*, *f*), *N*(*r*, *f*), *Q*(*r*, *f*), *T*(*r*, *f*), and *N* ^0^(*r*, 1/*f*′) instead of *M*_*α*,*β*_(*r*, *f*), *N*_*α*,*β*_(*r*, *f*), *Q*_*α*,*β*_(*r*, *f*), *T*_*α*,*β*_(*r*, *f*), and *N*_*α*,*β*_^0^(*r*, 1/*f*′), respectively.

By a similar discussion as in [23], one can obtain a standard and Valiron-Mohonko type result in *Ω* as follows.

**Lemma 16 (also see [16, Theorem 2.3.1])**

Let *f*(*z*) be a meromorphic function in *Ω*(*α*, *β*). Then for all irreducible rational function *R*(*z*, *f*) in *f* with coefficients meromorphic and small with respect to *f* in *Ω*(*α*, *β*), one has

(16) $$\begin{array}{c} T\left(r,R\left(z,f\right)\right)=dT\left(r,f\right)+Q\left(r,f\right), \end{array}$$

where *Q*(*r*, *f*) is stated as in (13) and *d* is the degree of *R*(*z*, *f*) in *f*.

**Lemma 17**

Suppose *f* is a nonconstant meromorphic function in *Ω*. Then

(17) $$\begin{array}{c} N\left(r,\frac{1}{f^{\prime }}\right)\leq N\left(r,\frac{1}{f}\right)+\bar{N}\left(r,f\right)+Q\left(r,f\right)+O\left(1\right), \end{array}$$

where *Q*(*r*, *f*) is stated as in (13).

**Proof**

Since

(18) M(r,1f)≤M(r,1f′)+M(r,f′f)=M(r,1f′)+Q(r,f),

then from properties of *T*(*r*, *f*), we have

(19) T(r,f)−N(r,1f)≤T(r,f′)−N(r,1f′) +Q(r,f)+O(1);

that is,

(20) N(r,1f′)≤T(r,f′)−T(r,f)+N(r,1f) +Q(r,f)+O(1).

Since

(21) T(r,f′)=M(r,f′)+N(r,f′)≤M(r,f)+M(r,f′f)+N(r,f)+N¯(r,f)≤T(r,f)+N¯(r,f)+Q(r,f)+O(1),

then from (20) and (21), we can get the conclusion of this lemma.

Next, we will give two main lemmas of this paper as follows.

**Lemma 18**

Let *F* and *G* be transcendental meromorphic functions in *Ω* in Tsuji sence satisfying *E*(0, *Ω*, *F*) = *E*(0, *Ω*, *G*), and let *c*_1_, *c*_2_,…, *c*_*q*_ be *q*(≥2) distinct nonzero complex numbers. If

(22) limsup⁡r→∞,r∈I3N¯(r,F)+∑j=1qN¯(2)(r,1/(F−cj))+N¯(r,1/F′)T(r,F)  <q,limsup⁡r→∞,r∈I3N¯(r,G)+∑j=1qN¯(2)(r,1/(G−cj))+N¯(r,1/G′)T(r,G)  <q,

where ${\bar{N}}^{\left(2\right)}\left(r,\cdot \right)=\bar{N}\left(r,\cdot \right)+{\bar{N}}^{(2}\left(r,\cdot \right)$, ${\bar{N}}^{(2}(r,\cdot )=\bar{N}(r,\cdot )-N^{1)}\left(r,\cdot \right)$, *N* ^1)^(*r*, ·) is the counting function which only counts simple zeros of the function · in *Ξ*(*α*, *β*; *r*), and *I* is some set of *r* of infinite linear measure, then

(23) $$\begin{array}{c} F=\frac{aG+b}{cG+d}, \end{array}$$

where *a*, *b*, *c*, *d* ∈ *C* are constants with *ad* − *bc* ≠ 0.

**Proof**

Set

(24) $$\begin{array}{c} H\equiv \frac{F^{\prime \prime }}{F^{\prime }}-2\frac{F^{\prime }}{F}-\left(\frac{G^{\prime \prime }}{G^{\prime }}-2\frac{G^{\prime }}{G}\right). \end{array}$$

Suppose that *H*≢0; from Lemma 15 and (13), we have

(25) $$\begin{array}{c} M\left(r,H\right)=Q\left(r\right), \end{array}$$

where *Q*(*r*) : = *o*{*T*(*r*)} and *T*(*r*) = max⁡{*T*(*r*, *F*), *T*(*r*, *G*)}. Since *E*(0, *Ω*, *F*) = *E*(0, *Ω*, *G*) and by an elementary calculation, we can conclude that if *z*_0_ is a common simple zero of *F* and *G* in *Ω*, then *H*(*z*_0_) = 0. Thus, from (11), we have

(26) $$\begin{array}{c} N^{1)}\left(r\right)\leq N\left(r,\frac{1}{H}\right)\leq T\left(r,H\right)+O\left(1\right)\leq N\left(r,H\right)+Q\left(r\right), \end{array}$$

where *N* ^1)^(*r*) = *N* ^1)^(*r*, 1/*F*) = *N* ^1)^(*r*, 1/*G*). The poles of *H* in *Ω* can only occur at zeros of *F*′ and *G*′ in *Ω* or poles of *F* and *G* in *Ω*. Moreover, *H* only has simple zeros in *Ω*. Hence, from (26), we have

(27)   N1)(r)≤N¯(r,F)+N¯(r,G)+N¯0(r,1F′)+N¯0(r,1G′)+∑j=1qN¯(2(r,1F−cj)+∑j=1qN¯(2(r,1G−cj)+Q(r),

where ${\bar{N}}^{0}\left(r,1/F^{\prime }\right)$ is the reduced counting function for the zeros of *F*′ in *Ω* where *F* does not take one of the values 0, *c*_1_, *c*_2_,…, *c*_*q*_.

Since

(28) N¯(r,1F)+N¯(r,1G)=2N1)(r)+N¯(2(r,1F)+N¯(2(r,1G),

then from (27) and (28), we have

(29) N¯(r,1F)+N¯(r,1G) ≤2N¯(r,F)+2N¯(r,G)+2N¯0(r,1F′)+2N¯0(r,1G′)  +N¯(2(r,1F)+N¯(2(r,1G)+2∑j=1qN¯(2(r,1F−cj)  +2∑j=1qN¯(2(r,1G−cj)+Q(r).

From Remark 14, we have

(30) qT(r,F)≤N¯(r,F)+N¯(r,1F)+∑j=1qN¯(r,1F−cj) −N0(r,1F′)+Q(r), r∉E,qT(r,G)≤N¯(r,G)+N¯(r,1G)+∑j=1qN¯(r,1G−cj) −N0(r,1G′)+Q(r), r∉E,

where *E* is a set of *r* of finite linear measure, and it need not be the same at each occurrence. From (29)-(30), it follows for *r* ∉ *E* that

(31) q{T(r,F)+T(r,G)} ≤3N¯(r,F)+3N¯(r,G)+∑j=1qN¯(r,1F−cj)  +∑j=1qN¯(r,1G−cj)+2∑j=1qN¯(2(r,1F−cj)  +2∑j=1qN¯(2(r,1G−cj)+N¯0(r,1F′)+N¯0(r,1G′)  +N¯(2(r,1F)+N¯(2(r,1G)+Q(r),

since

(32) $$\begin{array}{c} \overset{q}{\underset{j=1}{\sum }}{\bar{N}}^{(2}\left(r,\frac{1}{F-c_{j}}\right)+{\bar{N}}^{(2}\left(r,\frac{1}{F}\right)+{\bar{N}}^{0}\left(r,\frac{1}{F^{\prime }}\right)=\bar{N}\left(r,\frac{1}{F^{\prime }}\right),\\ \overset{q}{\underset{j=1}{\sum }}{\bar{N}}^{(2}\left(r,\frac{1}{G-c_{j}}\right)+{\bar{N}}^{(2}\left(r,\frac{1}{G}\right)+{\bar{N}}^{0}\left(r,\frac{1}{G^{\prime }}\right)=\bar{N}\left(r,\frac{1}{G^{\prime }}\right). \end{array}$$

From (31)-(32), we have, for *r* ∉ *E*,

(33) q{T(r,F)+T(r,G)} ≤3N¯(r,F)+3N¯(r,G)+∑j=1qN¯(2)(r,1F−cj)  +∑j=1qN¯(2)(r,1G−cj)+N¯(r,1F′)+N¯(r,1G′)+Q(r).

From (22) and (33), since *F*, *G* are transcendental in Tsuji sense in *Ω*, we have

(34) $$\begin{array}{c} T\left(r,F\right)+T\left(r,G\right)\leq o\left\{T\left(r,F\right)+T\left(r,G\right)\right\},\;r\notin E,\;\;r\in I. \end{array}$$

Thus, we can get a contradiction. Therefore, *H*(*z*) ≡ 0; that is,

(35) $$\begin{array}{c} \frac{F^{\prime \prime }}{F^{\prime }}-2\frac{F^{\prime }}{F}\equiv \frac{G^{\prime \prime }}{G^{\prime }}-2\frac{G^{\prime }}{G}. \end{array}$$

For the above equality, by integration, it follows that

(36) $$\begin{array}{c} F\equiv \frac{aG+b}{cG+d}, \end{array}$$

where *a*, *b*, *c*, *d* ∈ *C* and *ad* − *bc* ≠ 0.

**Lemma 19**

Let *F* and *G* be meromorphic functions in *Ω* and transcendental in Tsuji sense, if *F* and *G* satisfy *E*_1_(0, *Ω*, *F*) = *E*_1_(0, *Ω*, *G*), and let *c*_1_, *c*_2_,…, *c*_*q*_ be *q*(≥2) distinct nonzero complex numbers. If

(37) limsup⁡r→∞,r∈I(3N¯(r,F)+∑j=1qN¯(2)(r,1F−cj)+N¯(r,1F′)+2N¯(2(r,1F))(T(r,F))−1<q,limsup⁡r→∞,r∈I(3N¯(r,G)+∑j=1qN¯(2)(r,1G−cj)+N¯(r,1G′)+2N¯(2(r,1G))(T(r,G))−1<q,

where ${\bar{N}}^{\left(2\right)}\left(r,\cdot \right)$, ${\bar{N}}^{(2}\left(r,\cdot \right)$, and *I* are stated as in Lemma 18, then

(38) $$\begin{array}{c} F=\frac{aG+b}{cG+d}, \end{array}$$

where *a*, *b*, *c*, *d* ∈ *C* are constants with *ad* − *bc* ≠ 0.

**Proof**

Let *H* be stated as in the proof of Lemma 18; since *E*_1_(0, *Ω*, *F*) = *E*_1_(0, *Ω*, *G*), it follows that

(39) N1)(r)≤N¯(r,F)+N¯(r,G)+N¯0(r,1F′)+N¯0(r,1G′) +N¯(2(r,1F)+N¯(2(r,1G)+∑j=1qN¯(2(r,1F−cj) +∑j=1qN¯(2(r,1G−cj).

Similar to argument as in Lemma 18, we have, for *r* ∉ *E*,

(40) q{T(r,F)+T(r,G)} ≤3N¯(r,F)+3N¯(r,G)+∑j=1qN¯(2)(r,1F−cj)  +∑j=1qN¯(2)(r,1G−cj)+2N¯(2(r,1F)+2N¯0(2(r,1G)  +N¯(r,1F′)+N¯(r,1G′)+Q(r).

From (37) and (40), since *f*, *g* are transcendental in Tsuji sense in *Ω*, it follows that

(41) $$\begin{array}{c} T\left(r,F\right)+T\left(r,G\right)\leq o\left\{T\left(r,F\right)+T\left(r,G\right)\right\},\;r\notin E,\;\;r\in I. \end{array}$$

Thus, we can get a contradiction. Therefore, *H*(*z*) ≡ 0; that is,

(42) $$\begin{array}{c} \frac{F^{\prime \prime }}{F^{\prime }}-2\frac{F^{\prime }}{F}\equiv \frac{G^{\prime \prime }}{G^{\prime }}-2\frac{G^{\prime }}{G}. \end{array}$$

For the above equality, by integration, we have

(43) $$\begin{array}{c} F\equiv \frac{aG+b}{cG+d}, \end{array}$$

where *a*, *b*, *c*, *d* ∈ *C* and *ad* − *bc* ≠ 0.

The following result can be derived from the proof of Frank-Reinders' theorem in [24].

**Lemma 20**

Let *n* ≥ 6 and

(44) $$\begin{array}{c} P\left(w\right)=\frac{\left(n-1\right)\left(n-2\right)}{2}w^{n}-n\left(n-2\right)w^{n-1}+\frac{n\left(n-1\right)}{2}w^{n-2}. \end{array}$$

Then *P*(*w*) is a unique polynomial for transcendental meromorphic functions; that is, for any two transcendental meromorphic functions *f* and *g* in Tsuji sense, *P*(*f*) ≡ *P*(*g*) implies *f* ≡ *g*.

**Lemma 21 (see [16, Lemma 2.3.3])**

Let *f*(*z*) be a meromorphic function in *Ω*(*α*, *β*), for any real number *ε* > 0, *Ω*_*ε*_ = *Ω*(*α* + *ε*, *β* − *ε*). Then for *ε* > 0, one has

(45) $$\begin{array}{c} N\left(r,f\right)\leq \omega \frac{N\left(r,\Omega ,f\right)}{r^{\omega }}+\omega^{2}\overset{r}{\underset{1}{\int }}\frac{N\left(t,\Omega ,f\right)}{t^{\omega +1}}\;dt,\\ N\left(r,f\right)\geq \omega c^{\omega }\frac{N\left(cr,\Omega_{\varepsilon },f\right)}{r^{\omega }}+\omega^{2}c^{\omega }\overset{cr}{\underset{1}{\int }}\frac{N\left(t,\Omega_{\varepsilon },f\right)}{t^{\omega +1}}\;dt, \end{array}$$

where 0 < *c* < 1 is a constant depending on *ε*, *ω* = (*π*/(*β* − *α*)), *N*(*t*, *Ω*, *f*) = ∫_1_^*r*^(*n*(*t*, *Ω*, *f*)/*t*)*dt*, and *n*(*t*, *Ω*, *f*) is the number of poles of *f*(*z*) in *Ω*∩{*z* : 1 < |*z*| ≤ *t*}.

## 4. Proofs of Theorems 5 and 7

### 4.1. The Proof of Theorem 5

From the definition of *P*_1_(*w*), we have *P*_1_(1) = 1 − *c* : = *c*_1_ ≠ 0, *P*_1_(0) = −*c* : = *c*_2_ ≠ 0, and

(46) $$\begin{array}{c} P_{1}^{\prime }\left(w\right)=\frac{n\left(n-1\right)\left(n-2\right)}{2}{\left(w-1\right)}^{2}w^{n-3}, \end{array}$$

(47) $$\begin{array}{c} P_{1}\left(w\right)-c_{1}={\left(w-1\right)}^{3}Q_{1}\left(w\right),\;Q_{1}\left(1\right)\neq 0, \end{array}$$

(48) $$\begin{array}{c} P_{1}\left(w\right)-c_{2}=w^{n-2}Q_{2}\left(w\right),\;Q_{2}\left(0\right)\neq 0, \end{array}$$

where *Q*_1_, *Q*_2_ are polynomials of degrees *n* − 3 and 2, respectively. We also see that *Q*_*i*_  (*i* = 1,2) and *P*_1_ have only simple zeros.

Let *F* and *G* be defined as *F* = *P*_1_(*f*) and *G* = *P*_1_(*g*). Since *E*(*S*, *Ω*, *f*) = *E*(*S*, *Ω*, *g*), we have *E*(0, *Ω*, *F*) = *E*(0, *Ω*, *G*). From (47) and (48), we have

(49) N¯(2)(r,1F−c1)=N¯(r,1F−c1)+N¯(2(r,1F−c1)≤2N¯(r,1f−1)+∑i=1n−3N(r,1f−ai)≤(n−1)T(r,f)+Q(r),N¯(2)(r,1F−c2)=N¯(r,1F−c2)+N¯(2(r,1F−c2)≤2N¯(r,1f)+∑j=12N(r,1f−bj)≤4T(r,f)+Q(r),

where *a*_*i*_  (*i* = 1,…, *n* − 3) and *b*_*j*_  (*j* = 1,2) are the zeros of *Q*_1_(*w*) and *Q*_2_(*w*) in *Ω*, respectively.

From (46), we have

(50) $$\begin{array}{c} \bar{N}\left(r,\frac{1}{F^{\prime }}\right)\leq \bar{N}\left(r,\frac{1}{f}\right)+\bar{N}\left(r,\frac{1}{f-1}\right)+\bar{N}\left(r,\frac{1}{f^{\prime }}\right). \end{array}$$

From Lemma 16, we have *T*(*r*, *F*) = *nT*(*r*, *f*) + *Q*(*r*). Thus, combining (49) and (50), by Lemmas 17 and 18 and *n* ≥ 11, we have

(51) limsup⁡r→∞,r∉E3N¯(r,F)+∑j=12N¯(2)(r,1/(F−cj))+N¯(r,1/F′)T(r,F) ≤limsup⁡r→∞,r∉E4N¯(r,f)+(n+6)T(r,f)nT(r,f)<2.

Similarly, we can obtain

(52) limsup⁡r→∞,r∉E3N¯(r,G)+∑j=12N¯(2)(r,1/(G−cj))+N¯(r,1/G′)T(r,G) ≤limsup⁡r→∞,r∉E4N¯(r,g)+(n+6)T(r,g)nT(r,g)<2.

Thus, by Lemma 18, we have

(53) $$\begin{array}{c} \frac{F^{\prime \prime }}{F^{\prime }}-2\frac{F^{\prime }}{F}\equiv \frac{G^{\prime \prime }}{G^{\prime }}-2\frac{G^{\prime }}{G}. \end{array}$$

For the above equality, by integration, it follows that

(54) $$\begin{array}{c} F\equiv \frac{aG+b}{cG+d}, \end{array}$$

where *a*, *b*, *c*, *d* ∈ *C* and *ad* − *bc* ≠ 0. Since *E*(*f*, *Ω*, *S*) is nonempty and *E*(*f*, *Ω*, *S*) = *E*(*g*, *Ω*, *S*), we have *b* = 0, *a* ≠ 0. Hence

(55) $$\begin{array}{c} F\equiv \frac{aG}{cG+d}\equiv \frac{G}{AG+B}, \end{array}$$

where *A* = *c*/*a*, *B* = *d*/*a* ≠ 0.

Two cases will be considered as follows.

**Case 1 (*A* ≠ 0)**

From the definition of *P*_1_(*w*) and (55), we can see that every zero of *P*_1_(*g*) + *B*/*A* in *Ω* has a multiplicity of at least *n*. Here, three following subcases will be discussed.

**Subcase 1 (*B*/*A* = −*c*_1_)**

From (47), we have

(56) $$\begin{array}{c} P_{1}\left(g\right)+\frac{B}{A}={\left(g-1\right)}^{3}\left(g-a_{1}\right)\left(g-a_{2}\right)\cdots \left(g-a_{n-3}\right), \end{array}$$

where *a*_*i*_ ≠ 0,1 are distinct values. It follows that

(57) $$\begin{array}{c} \Theta_{T}\left(a_{i},f\right)=1-\underset{r\to \infty }{\mathrm{limsup}}\frac{\bar{N}\left(r,a\right)}{T\left(r,f\right)}\geq 1-\underset{r\to \infty }{\mathrm{limsup}}\frac{\bar{N}\left(r,a\right)}{N\left(r,f\right)}\geq \frac{1}{2}. \end{array}$$

We can see that it has *n* − 2 values satisfying the above inequality. Thus, from (21) and *n* ≥ 11, we can get a contradiction.

**Subcase 2 (*B*/*A* = −*c*_2_)**

From (47), we have

(58) $$\begin{array}{c} P_{1}\left(g\right)+\frac{B}{A}=g^{n-2}\left(g-b_{1}\right)\left(g-b_{2}\right), \end{array}$$

where *b*_1_ ≠ *b*_2_, *b*_*i*_ ≠ 0,1  (*i* = 1,2). It follows that every zero of *g* in *Ω* has a multiplicity of at least 2 and every zero of *g* − *b*_*i*_  (*i* = 1,2) in *Ω* has a multiplicity of at least *n*. Then, by Remark 14, we have

(59) T(r,g) ≤N¯(r,1g)+N¯(r,1g−b1)+N¯(r,1g−b2)+Q(r) ≤12N(r,1g)+1nN(r,1g−b1)+1nN(r,1g−b1)+Q(r) ≤(12+2n)T(r,g)+Q(r).

Since *g* is transcendental in Tsuji sense in *Ω* and *n* ≥ 11, we can get a contradiction.

**Subcase 3 (*B*/*A* ≠ −*c*_1_, −*c*_2_)**

By using the same argument as in Subcase 1 or Subcase 2, we can get a contradiction.

**Case 2 (*A* = 0)**

If *B* ≠ 1, from (55), we have *F* = *G*/*B*; that is,

(60) $$\begin{array}{c} P_{1}\left(f\right)=\frac{1}{B}P_{1}\left(g\right). \end{array}$$

From (48) and (60), we have

(61) $$\begin{array}{c} P_{1}\left(f\right)-\frac{c_{2}}{B}=\frac{1}{B}\left(P_{1}\left(g\right)-c_{2}\right)=\frac{1}{B}g^{n-2}\left(g-b_{1}\right)\left(g-b_{2}\right). \end{array}$$

Since *c*_2_/*B* ≠ *c*_2_, from (46), it follows that *P*_1_(*f*) − *c*_2_/*B* has at least *n* − 2 distinct zeros  *e*_1_, *e*_2_,…, *e*_*n*−2_. Then, by Remark 14, we have

(62) (n−4)T(r,f) ≤∑i=1n−2N¯(r,1f−ei)+Q(r) ≤N¯(r,1g)+N¯(r,1g−b1)+N¯(r,1g−b2)+Q(r) ≤3T(r,g)+Q(r).

By applying Lemma 18 to (60) and from (62), since *n* ≥ 11 and *f* is transcendental in Tsuji sense in *Ω*, we can get a contradiction.

Thus, we have *A* = 0 and *B* = 1; that is, *P*_1_(*f*) = *P*_1_(*g*). Notting the form of *P*_1_(*w*), we can get that *P*(*f*) = *P*(*g*). Then, by Lemma 20, we get *f* ≡ *g*.

Therefore, the proof of Theorem 5 is completed.

### 4.2. The Proof of Theorem 7

Since Θ_*T*_(*∞*, *f*) > 3/4 and Θ_*T*_(*∞*, *g*) > 3/4, it follows that

(63) $$\begin{array}{c} \underset{r\to \infty }{\mathrm{limsup}}\frac{\bar{N}\left(r,f\right)}{T\left(r,f\right)}<\frac{1}{4},\;\;\underset{r\to \infty }{\mathrm{limsup}}\frac{\bar{N}\left(r,g\right)}{T\left(r,g\right)}<\frac{1}{4}. \end{array}$$

By applying (60), from (51) and (52), since *n* ≥ 7, we get

(64) limsup⁡r→∞,r∉E3N¯(r,F)+∑j=12N¯(2)(r,1/(F−cj))+N¯(r,1/F′)T(r,F) ≤limsup⁡r→∞,r∉E4N¯(r,f)+(n+6)T(r,f)nT(r,f)<2,limsup⁡r→∞,r∉E3N¯(r,G)+∑j=12N¯(2)(r,1/(G−cj))+N¯(r,1/G′)T(r,G) ≤limsup⁡r→∞,r∉E4N¯(r,g)+(n+6)T(r,g)nT(r,g)<2.

Then, from Lemma 18, we have *F* ≡ (*aG* + *b*)/(*cG* + *d*), where *a*, *b*, *c*, *d* ∈ *C* and *ad* − *bc* ≠ 0. Thus, by using the same argument as in Theorem 5, we can prove the conclusion of Theorem 7.

## 5. Proofs of Theorems 9 and 11

### 5.1. The Proof of Theorem 9

Since *E*_1_(*S*, *Ω*, *f*) = *E*_1_(*S*, *Ω*, *g*), we have *E*_1_(0, *Ω*, *F*) = *E*_1_(0, *Ω*, *G*). From (46)–(48), we get

(65) $$\begin{array}{c} {\bar{N}}^{(2}\left(r,\frac{1}{F}\right)=\overset{n}{\underset{i=1}{\sum }}\bar{N}\left(r,\frac{1}{f-d_{i}}\right)\leq \bar{N}\left(r,\frac{1}{f^{\prime }}\right), \end{array}$$

where *d*_*i*_  (*i* = 1,…, *n*) are the distinct zeros of *P*_1_(*w*). And from (50) and (65), by Lemma 17, we have

(66) N¯0(r,1F′)+2N¯(2(r,1F) ≤N¯(r,1f)+N¯(r,1f−1)+3N(r,1f)+3N¯(r,f) ≤5T(r,f)+3N¯(r,f)+Q(r).

Then from (49) and (66), since *T*(*r*, *F*) = *nT*(*r*, *f*) + *Q*(*r*) and *n* ≥ 15, we have

(67) limsup⁡r→∞,r∉E(3N¯(r,F)+∑j=12N¯(2)(r,1F−cj)+N¯(r,1F′)  +2N¯(2(r,1F))(T(r,F))−1 ≤limsup⁡r→∞,r∉E6N¯(r,f)+(n+8)T(r,f)nT(r,f)<2.

Similarly, we get

(68) limsup⁡r→∞,r∉E(3N¯(r,G)+∑j=12N¯(2)(r,1G−cj)+N¯(r,1G′)+2N¯(2(r,1G))(T(r,G))−1 ≤limsup⁡r→∞,r∉E6N¯(r,g)+(n+8)T(r,g)nT(r,g)<2.

Thus, by Lemma 19, we have

(69) $$\begin{array}{c} F\equiv \frac{aG+b}{cG+d}, \end{array}$$

where *a*, *b*, *c*, *d* ∈ *C* and *ad* − *bc* ≠ 0. By using arguments similar to that in proof of Theorem 5, we have *f* ≡ *g*.

Therefore, this completes the proof of Theorem 9.

### 5.2. The Proof of Theorem 11

Since Θ_*T*_(*∞*, *f*) > 5/6 and Θ_*T*_(*∞*, *g*) > 5/6, it follows that

(70) $$\begin{array}{c} \underset{r\to \infty }{\mathrm{limsup}}\frac{\bar{N}\left(r,f\right)}{T\left(r,f\right)}<\frac{1}{6},\;\;\underset{R\to \infty }{\mathrm{limsup}}\frac{\bar{N}\left(r,g\right)}{T\left(r,g\right)}<\frac{1}{6}. \end{array}$$

By applying (70), from (67) and (68), since *n* ≥ 9, we get

(71) limsup⁡r→∞,r∉E(3N¯(r,F)+∑j=12N¯(2)(r,1F−cj)+N¯(r,1F′)+2N¯(2(r,1F))(T(r,F))−1 ≤limsup⁡r→∞,r∉E6N¯(r,f)+(n+8)T(r,f)nT(r,f)<2,

(72) limsup⁡r→∞,r∉E(3N¯(r,G)+∑j=12N¯(2)(r,1G−cj)+N¯(r,1G′)+2N¯(2(r,1G))(T(r,G))−1 ≤limsup⁡r→∞,r∉E6N¯(r,g)+(n+8)T(r,g)nT(r,g)<2.

Then, from Lemma 19, we have *F* ≡ (*aG* + *b*)/(*cG* + *d*), where *a*, *b*, *c*, *d* ∈ *C* and *ad* − *bc* ≠ 0. Thus, by using the same argument as in Theorem 5, we can prove the conclusion of Theorem 11.

Hence, the proof of Theorem 11 is completed.

## 6. The proof of Theorem 13

Since condition ((‡)) implies that *f* is transcendental in Tsuji sense, then the conclusions of Theorem 13 can be obtained easily from Theorems 5–11 and Corollaries 6–12.

## References

[1] Hayman W (1964). *Meromorphic Functions*.

[2] Yi HX, Yang CC (2003). *Uniqueness Theory of Meromorphic Functions*.

[3] Nevanlinna R (1926). Einige Eindeutigkeitssätze in der Theorie der Meromorphen Funktionen. *Acta Mathematica*.

[4] Zheng J-H (2004). On uniqueness of meromorphic functions with shared values in some angular domains. *Canadian Mathematical Bulletin*.

[5] Gol’dberg AA (1975). Nevanlinna’s lemma on the logarithmic derivative of a meromorphic function. *Mathematical Notes of the Academy of Sciences of the USSR*.

[6] Gol'dberg AA, Ostrovskii IV (1970). *The Distribution of Values of Meromorphic Function*.

[7] Lin W, Mori S, Tohge K (2006). Uniqueness theorems in an angular domain. *Tohoku Mathematical Journal*.

[8] Lin WC, Mori S, Yi HX (2008). Uniqueness theorems of entire functions with shared-set in an angular domain. *Acta Mathematica Sinica*.

[9] Long JR, Wu PC (2012). Borel directions and uniqueness of meromorphic functions. *Chinese Annals of Mathematics*.

[10] Wu Z-J, Sun D-C (2008). A remark on uniqueness theorems in an angular domain. *Proceedings of the Japan Academy Series A: Mathematical Sciences*.

[11] Xu H-Y, Cao T-B (2010). Uniqueness of meromorphic functions sharing four values im and one set in an angular domain. *Bulletin of the Belgian Mathematical Society-Simon Stevin*.

[12] Xu HY, Wu ZJ, Tu J (2014). Some inequalities and applications on Borel direction and exceptional values of meromorphic functions. *Journal of Inequalities and Applications*.

[13] Zhang KY, Xu HY, Yi HX (2013). Borel directions and uniqueness of meromorphic functions. *Abstract and Applied Analysis*.

[14] Zhang QC (2013). Borel's directions and shared values. *Acta Mathematica Scientia B*.

[15] Zhang Q (2009). Meromorphic functions sharing values in an angular domain. *Journal of Mathematical Analysis and Applications*.

[16] Zheng JH (2010). *Value Distrbution of Meromorphic Functions*.

[17] Zheng JH (2003). On uniqueness of meromorphic functions with shared values in one angular domains. *Complex Variables and Elliptic Equations*.

[18] Cao T-B, Yi H-X (2009). On the uniqueness of meromorphic functions that share four values in one angular domain. *Journal of Mathematical Analysis and Applications*.

[19] Xu J, Yi H (2008). On uniqueness of meromorphic functions with shared four values in some angular domains. *Bulletin of the Malaysian Mathematical Sciences Society*.

[20] Xuan Z-X (2009). On uniqueness of meromorphic functions with multiple values in some angular domains. *Journal of Inequalities and Applications*.

[21] Chen S, Lin W (2011). On uniqueness of Meromorphicfunctions with shared-sets in an angular domain. *Acta Mathematica Scientia*.

[22] Langley JK (1991). An application of the Tsuji characteristic. *Journal of The Faculty of Science, The University of Tokyo, Section IA, Mathematics*.

[23] Mokhon'ko AZ (1971). The Nevanlinna characteristics of some meromorphic functions. *Functional Analysis and Their Applications*.

[24] Frank G, Reinders M (1998). A uniqueness range sets for meromorphic functions with 11 elements. *Complex Variables, Theory and Application*.

